# Ectopic ACTH syndrome of different origin—Diagnostic approach and clinical outcome. Experience of one Clinical Centre

**DOI:** 10.1371/journal.pone.0242679

**Published:** 2020-11-25

**Authors:** Joanna Ewelina Paleń-Tytko, Elwira Maria Przybylik-Mazurek, Ewelina Joanna Rzepka, Dorota Magdalena Pach, Anna Stanisława Sowa-Staszczak, Aleksandra Gilis-Januszewska, Alicja Bronisława Hubalewska-Dydejczyk

**Affiliations:** Department of Endocrinology, Jagiellonian University, Krakow, Poland; Augusta University, UNITED STATES

## Abstract

**Purpose:**

Ectopic Cushing Syndrome (EAS) is a rare condition responsible for about 5–20% of all Cushing syndrome cases. It increases the mortality of affected patients thus finding and removal of the ACTH-producing source allows for curing or reduction of symptoms and serum cortisol levels. The aim of this study is to present a 20-year experience in the diagnosis and clinical course of patients with EAS in a single Clinical Centre in Southern Poland as well as a comparison of clinical course and outcomes depending on the source of ectopic ACTH production–especially neuroendocrine tumors with other neoplasms.

**Methods:**

Twenty-four patients were involved in the clinical study with EAS diagnosed at the Department of Endocrinology between years 2000 and 2018. The diagnosis of EAS was based on the clinical presentation, hypercortisolemia with high ACTH levels, high dose dexamethasone suppression test and/or corticotropin-releasing hormone tests. To find the source of ACTH various imaging studies were performed.

**Results:**

Half of the patients were diagnosed with neuroendocrine tumors, whereby muscle weakness was the leading symptom. Typical cushingoid appearance was seen in merely a few patients, and weight loss was more common than weight gain. Patients with neuroendocrine tumors had significantly higher midnight cortisol levels than the rest of the group. Among patients with infections, we observed a significantly higher concentrations of cortisol 2400 levels in gastroenteropancreatic neuroendocrine tumors. Chromogranin A correlated significantly with potassium in patients with neuroendocrine tumors and there was a significant correlation between ACTH level and severity of hypokalemia.

**Conclusion:**

EAS is not common, but if it occurs it increases the mortality of patients; therefore, it should be taken into consideration in the case of coexistence of severe hypokalemia with hypertension and muscle weakness, especially when weight loss occurs. Because the diagnosis of gastroenteropancreatic neuroendocrine tumor worsens the prognosis-special attention should be paid to these patients.

## Introduction

Hypercortisolemia and a set of symptoms caused by it is defined as Cushing Syndrome (CS). In most cases the source of CS lies in the excessive administration of glucocorticoids for various medical reasons [1]. As regards endogenous causes, they are divided into two groups: adrenocorticotropic hormone (ACTH)-dependent and ACTH-independent CS, responsible for about 70–80% and 20–30% of cases respectively [2]. Adenoma of the pituitary gland producing ACTH—Cushing disease (CD) is the most common source of endogenous hypercortisolemia, which accounts for about 60–70% of all CS cases [3] whereas less common are adenomas of the suprarenal gland—a condition known as ACTH-independent CS (10–20% of all CS patients) [2].

Ectopic Cushing Syndrome (EAS) is a rare condition, responsible for about 5–20% of all (CS) cases and ca. 10–20% of ACTH-dependent CS patients [2, 4–11]. The first time when EAS was named and largely studied was in the early 1960s by Liddle and soon after by Meador [12, 13]. It plays a pivotal role to extract the group of EAS patients from all CS patients due to a different management. Removal of the ACTH-producing source allows for curing or a significant reduction of symptoms and serum cortisol levels. It is crucial to be active in searching for the tumor that produces ACTH; some malignant, aggressive tumors could be hidden behind it and failure to recognize it could result in poor prognosis [8, 14, 15]. In most cases the source of ectopic production of ACTH is located in the lungs and mediastinum, but it can also be produced by tumors originating from other parts of the body, such as gastroenteropancreatic neuroendocrine tumors (GepNETs), pheochromocytomas and other [4].

The objective of this study is to present 20-year experience in the diagnosis and prognosis of patients with EAS in a single Clinical Centre in Southern Poland. To the best of our knowledge, this is the first work on the Polish population of patients with EAS, studying diagnostics and clinical course depending on the type of tumor producing ACTH. We aimed to analyze the course of EAS in NET and especially in GepNET patients, compared to other locations.

## Methods

We retrospectively reviewed the records of patients with EAS diagnosed at the Department of Endocrinology between 2000 and 2018 and reviewed the records for routine (but typical for CS) and endocrine biochemical tests: ACTH was measured by immunoradiometric assay (Brahms, Henningsdorf, Germany), whereas plasma cortisol was measured by electrochemiluminescence method (Roche Diagnostics GmbH, Mannheim, Germany).

To find the source of ACTH, biochemical parameters were checked (chromogranin A, calcitonin, 5-hydroxyindoleacetic acid (5-HIAA), urine metabolites of catecholamines)) carcinoembryonic antigen (CEA) and cancer antigens: Ca 125, Ca 19–9 Ca 15–3, with various imaging studies: anatomical: ultrasound of the abdomen (US), computed tomography (CT), magnetic resonance imaging (MRI) and functional: positron emission tomography with fludeoxyglucose (FDG-PET) and/or somatostatin receptor scintigraphy (SRS) were performed.

The diagnosis of EAS was based on the clinical presentation, hypercortisolemia with high ACTH levels, high-dose dexamethasone suppression test (HDDST) and/or corticotropin-releasing hormone (CRH) tests, because bilateral inferior petrosal sinus sampling (BIPSS) was not available—mostly due to poor condition of the patients. Based on imaging techniques (CT or MR), visible pituitary focal lesions were excluded as a cause of high level of ACTH.

### Statistical methods

Statistical analysis was performed using STATISTICA 13.1 software (StatSoft, Inc., Tulsa, USA). The data normality distribution was assessed using the Kruskal–Wallis test with the Lilliefors correction. Non–parametric tests were applied due to rejection of the normality hypothesis for most of the analyzed parameters. The level of significance for all tests was set at 0.05. The differences in median values were tested using the Mann–Whitney U test. Differences in the number of patients, categorized by any criteria defined in this study, were tested using a contingency table and the results were assessed based on a chi–square test with the Yates correction. The Kaplan–Meier plot and Cox proportional hazards model with FCox statistics were used to assess differences in mortality in patients dichotomized by any criteria in this study, where patients who were alive were classified as censored observations. The Spearman correlation was used to test the relationship between the parameters and logistic regression was used to prepare a model for predicting mortality. The selection of significant predictors was based on the probability of the likelihood–ratio statistic. Finally, statistical significance was assessed using the CHI^2^ test for the overall model and the Wald test statistics for the predictors.

### Ethics statement

The study was approved by the Bioethics Committee of the Jagiellonian University (reference no.: 1072.6120.213.2019) and was performed in accordance with the ethical standards as laid down in the 1964 Declaration of Helsinki and its later amendments or comparable ethical standards. Prior to performing any procedure, and after obtaining comprehensive information, each patient signed informed consent, which is included in the patient’s medical history.

## Results

Twenty-four patients with EAS were involved in the study: 14 women and 10 men (female to male ratio 1.4:1), with a median age at the time of the diagnosis being 61 years.

Persistent hypokalemia with high suspicion of hypercortisolism was the reason for referral to our Clinical Center in most of the cases. Merely 8 patients presented a typical cushingoid feature and one patient has previously been treated for Guillain-Barre syndrome (due to sudden onset of muscle weakness). Half of the patients were diagnosed with neuroendocrine neoplasms– 6 females and 6 males (GepNETs, thymic and pulmonary carcinoids); among non-NET patients, two were found with pheochromocytoma, one with esthesioneuroblastoma, two with medullary thyroid carcinoma, two with carcinoma of the ovary, while the remaining patients—with single small-cell lung carcinoma (SCLS), papilloma of the maxillary sinus and adenocarcinoma of the stomach. In two patients in terminal state, who were only palliative treated, although the potential source of ACTH was found (tumor of the pancreas and lung in imagining studies), tissue specimen for histopathological examination was not available. Table 1.

**Table 1:** Patients`characteristics.

Patient No.	Age (years)^1^	Gender	Tumor	Metastases	Outcome
1	32	F	Pancreatic NET	+	Deceased
2	42	M	Thymic carcinoid	+	Deceased
3	32	M	Pancreatic NEC	+	Deceased
4	55	M	SCLC	+	Deceased
5	60	M	Gastric NEC	+	Deceased
6	59	M	Pulmonary carcinoid	-	Alive
7	62	F	Ovarian carcinoma	+	Deceased
8	70	F	Pheochromocytoma	-	Deceased
9	44	M	Esthesioneuroblastoma	-	Alive
10	67	F	Pancreatic NEC	+	Deceased
11	38	M	Colon NET	na	Deceased
12	14	M	Medullary thyroid carcinoma	+	Deceased
13	76	F	Maxillary sinus papilloma	-	Alive
14	61	F	Pheochromocytoma	-	Alive
15	78	F	Ovarian carcinoma	+	Deceased
16	61	F	Pancreatic NET	+	Deceased
17	47	F	Pancreatic NET	+	Deceased
18	66	F	Pancreatic tumor ^2^	+	Deceased
19	85	F	Lung tumor^2^	-	Deceased
20	61	F	Pulmonary carcinoid	-	Alive
21	65	F	Gastric adenocarcinoma	+	Deceased
22	65	F	Pancreatic NET	+	Deceased
23	49	M	Medullary thyroid carcinoma	+	Alive
24	71	M	Ileum NET	+	Deceased

Abbreviations: F-female; M- male; na-not available; NET- neuroendocrine tumor; NEC- neuroendocrine carcinoma;

SCLC-small cell lung carcinoma; + positive;—negative.

^1^at the time of diagnosis

^2^ no histopathological specimen with staining for ACTH available

The most common clinical findings are shown in Table 2.

**Table 2:** Clinical symptoms.

		total group			female			Male		
Clinical features	n (% of patients) N = 24	NET n = 12 (% of patients)	non-NET n = 12 (% of patients)	p	NET n = 6 (% of patients)	non-NET n = 8 (% of patients)	p	NET n = 6 (% of patients)	non-NET n = 4 (% of patients)	p
Muscle weakness	21 (88)	10(83)	11(92)	n.s.	5(83)	8(100)	n.s.	5(83)	3(75)	n.s.
Hypertension	19 (79)	9(75)	10(83)	n.s.	5(83)	7(88)	n.s.	4(67)	3(75)	n.s.
Infections	16 (66)	9(75)	7(58)	n.s.	4(67)	4(50)	n.s.	5(83)	3(75)	n.s.
Peripheral oedema	15 (63)	10(91)	5(42)	0.041	6(100)	4(50)	n.s.	4(67)	1(25)	0.170
Facial plethora	13 (54)	8(67)	5(42)	n.s.	2(33)	3(38)	n.s.	6(100)	2(50)	n.s.
Easy bruising	13 (54)	9(75)	4(36)	n.s.	3(50)	6(75)	n.s.	3(50)	1(25)	n.s.
Redistribution of fat tissue	12 (50)	9(75)	3(25)	0.041	4(67)	2(25)	n.s.	5(83)	1(25)	n.s.
Weight loss	11 (45)	7 (64)	4(36)	n.s.	2(33)	4(50)	n.s.	2(33)	3(75)	n.s.
Psychiatric disorders	10 (42)	5(42)	5(50)	n.s.	0	3(38)	n.s.	5(83)	2(50)	n.s.
Overweight/Obesity	9 (38)	5(42)	4(36)	n.s.	3(50)	3(38)	n.s.	2(33)	1(25)	n.s.
Weight gain	7 (29)	4 (33)	3(25)	n.s.	1(17)	2(25)	n.s.	3(50)	1(25)	n.s.
Hirsutism^1^	6 (46)	---	---	---	4(67)	2(29)	n.s.	---	---	---
Striae	3 (13)	2(17)	1(8)	n.s.	0(0)	0(0)	---	2(33)	1(25)	n.s.

^1^only female patients

Abbreviations: NET- neuroendocrine tumor; n.s—non-statistically significant

There were no significant gender differences in clinical presentation, although there was a higher percentage of hypertension and peripheral oedema in females, while facial plethora and psychiatric disorders were more common in males. In general, muscle weakness was the leading symptom and typical cushingoid appearance (facial plethora, easy bruising, redistribution of fat tissue, weight gain and hirsutism) was present in merely a few patients, most likely in NET individuals. Weight loss was more prevalent than weight gain (11 vs 7 patients). Redistribution of the fat tissue characteristic for cushingoid appearance and peripheral oedema were considerably more common in NET patients (p = 0.041). Facial plethora was present in 13 patients (8 being NET patients) whereas easy bruising was seen in more than 50% of patients (in 13 of 24); in this group, 9 patients had NET. Clinical data on osteoporosis was available only in 9 cases: in 7 osteoporosis was diagnosed (in 5 patients with NET, and in single cases of carcinoma of the ovary and tumor of the lung). Diabetes or a pre-diabetic state were present in 75% of patients (18 of 24), 10 being NET patients.

As regarding infections—they were documented in 16 patients (7 GepNET, 1 pheochromocytoma, 1 esthesioneuroblastoma, 1 thymic carcinoid, 1 SCLC, 1 pulmonary carcinoid, 1 ovarian carcinoma, 1 medullary carcinoma, 1 papilloma and 1 lung tumor). 8 of them had an infection in more than 1 anatomic site. Table 3.

**Table 3:** Characteristics of infections in EAS patients.

Site	No. of patients	Number of patients with infection/ Pathogen
Respiratory	5	1.Klebsiella oxytoca, Candida albicans
		1.Klebsiella pneumonia only
		3. na
Gastrointestinal tract		
• oral cavity	2	1.Cryptoccocus laurentii,
		1.na
• esophagus	2	1.Candida albicans
		1. fungal infection
• small intestine	1	1.Rotavirus
• liver-AVH	1	1. Hepatitis B and C virus
Urinary tract	6	1.Enterococcus faecalis, Streptoccocus viridans
		1.Enterococcus faecalis VRE
		2.Escherichia coli
		1.Enterococcus faecalis HLAR, Escherichia coli, Candida albicans
		1.na
Ear/nose		
• otitis media	1	1.na
Central venous catheter	1	1.na
Cutaneous		
acne	1	1.na
Eye		
• bilateral fungal endophthalmitis	1	1.na
• bacterial conjunctivitis	1	1.na
Postoperative wound	3	1.Serratia marcescens, Morganella morgani
		1.Staphylococcus epidermidis
		1. Escherichia coli ESBL
Central nervous system	1	1.na
Other		
• coxitis fugax	1	1.na
• suprahepatic abscess	1	1.na
• abscess in the bursa omentalis	1	1.Enterobacter cloacae ESBL
• sepsis	2	1. Escherichia coli
		1.Klebasiella pneumoniae ESBL, Escherichia coli

Abbreviations: na- not available; VRE—Vancomycin-resistant enterococci, HLAR—High-level aminoglycoside resistance; ESBL—Extended spectrum beta-lactamases; AVH—Acute viral hepatitis

**Fig 1:**
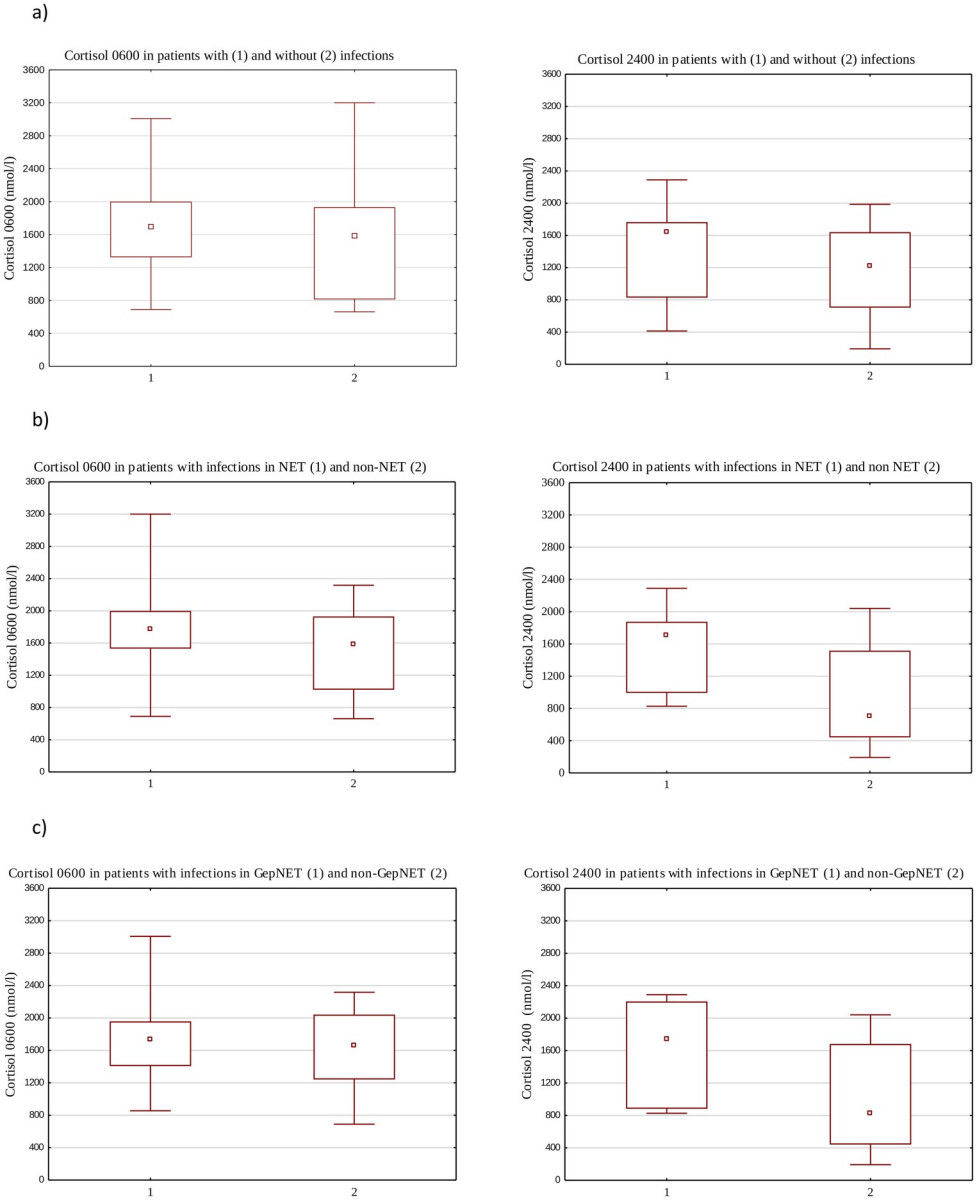
a. Cortisol 0600 and 2400 in patients with (1) and without (2) infections. b. Cortisol 0600 and 2400 in NET (1) and non-NET (2) patients with infections. c. Cortisol 0600 and 2400 in GepNET (1) and non-GepNET (2) patients with infections.

Patients with infections were treated based on the antibiogram results when available, in other cases- empiric therapy was applied in accordance to official and local antimicrobial policy. Individuals with infections had higher cortisol 0600 and 2400 concentrations than those without it, although this did not reach statistical significance (p>0.05), what is more, among patients with infections, we observed significantly higher concentrations of cortisol 2400 levels in NET (p = 0.002) and GepNET (p = 0.046) patients. Fig 1a–1c.

Table 4 presents the most essential laboratory evaluations.

**Table 4:** Data of all EAS patients.

	Total group		Female		Male	
Parameter /reference range	Med(Q1-Q3)	Range	Med(Q1-Q3)	Range	Med(Q1-Q3)	Range
**K** /3.50–5.10 mmol/l	2.65 (2.02–3.67)	1.61–4.90	2.45 (1.95–3.42)	1.61–4.90	2.81 (2.43–4.24)	1.70–4.62
**Na** /136.00–145.00 mmol/l	145.00 (142.00–148.93)	135.00–153.00	145.00 (141.50–149.25)	135.00–153.00	145.10 (142.00–149.03)	138.00–151.00
**Ca** /2.15–2.55 mmol/l	2.12 (1.94–2.31)	1.37–3.18	2.20 (2.04–2.32)	1.65–2.42	2.00 (1.92–2.29)	1.37–3.18
**P** /0.81–1.45 mmol/l	0.94 (0.69–1.18)	0.42–1.86	0.94 (0.69–1.16)	0.42–1.53	0.92 (0.66–1.26)	0.59–1.86
**TSH** /0.27–4.20 mIU/ml	0.36 (0.14–0.70)	0.09–3.23	0.32 (0.08–0.75)	0.09–3.23	0.36 (0.17–0.63)	0.06–0.70
**cortisol 0600** /63.45–642.76 nmol/l	1655.17 (1200.00–2000.00)	662.07–3200.00	1627.59 (1241.38–1958.62)	744.83–3200.00	1737.93 (855.17–2041.38)	662.07–3006.90
**cortisol 2400** /<50 nmol/l	1434.48 (717.24–1765.52)	193.10–2289.65	1227.59 (551.72–1765.52)	193.10–2206.90	1710.34 (855.18–1862.07)	717.24–2289.65
**cortisol 0600 h:2400 h ratio** />2.0	1.09 (1.09–1.73)	0.83–4.63	1.48 (1.04–2.39)	0.88–4.63	0.98 (0.93–1.10)	0.83–1.31
**ACTH** /1.32–12.33 pmol/l	70.40 (34.10–88.40)	14.74–312.62	66.00 (36.30–88.66)	14.70–153.56	70.40 (29.48–86.68)	15.40–312.62

Abbreviations: Med-median; Q1-first quartile; Q3-third quartile; range-minimum to maximum range; cortisol 0600- early morning cortisol; cortisol 2400- midnight cortisol ACTH—adrenocorticotropic hormone; K—potassium; Na—sodium; Ca—calcium; P—phosphate; TSH—thyroid-stimulating hormone.

Not surprisingly, most of the patients had low TSH levels with a median concentration of 0.36 mIU/ml (in 21 of 24 it was lower than 1.0mIU/ml, 1 patient had TSH level of 3.23mIU/ml, for the remaining 2 there was no data) and he range of sodium (Na) concentrations varied from 135mmol/l up to 153 mmol/l (median—145mmol/l). In 15 patients from our group, we measured the acid-base balance. Among 10 patients with metabolic alkalosis, 9 were hypokalemic, while on the other hand, correct acid-base balance was observed only in normokalemic patients.

All subjects had elevated morning serum cortisol levels and midnight plasma cortisol levels, with a median morning and midnight plasma cortisol level 1655.17 nmol/l and 1434.48 nmol/l respectively. Twenty of 24 patients had lost their cortisol circadian rhythm, whereby of the remaining 4, all were non-NET female patients, with lung tumor, papilloma, gastric adenocarcinoma, and ovarian carcinoma. The range of basal plasma ACTH levels varied from 14.74 up to 312.62 pmol/l, with median 70.4 pmol/l (all were above the upper limit; with a reference range 1.32–12.33pmol/l). All patients showed an incorrect response in dynamic tests (LDDST, HDDST, CRH test). Table 4 also shows that the majority of patients suffered from hypokalemia with median potassium (K) concentrations 2.65mmol/l, which affected 17 of 24 individuals (70%; all but one had potassium levels lower than 3.0 mmol/l). However, all patients had an evidence of prior hypokalemia in medical history. Furthermore, there was a significant correlation between ACTH level and severity of hypokalemia (p<0.05) despite the source of EAS. Table 5.

**Table 5:** Correlation between ACTH, cortisol, chromogranin A and electrolytes in EAS patients.

	*electrolyte*	NET		non-NET	
		n = 12		n = 12	
		R Spearman	p-value	R Spearman	p-value
ACTH	Na	n.s.		0.830	< 0.001
	K	–0.606	0.037	–0.634	0.027
	P	n.s.		–0.606	0.048
cortisol 0600	K	n.s.	-0.697	0.025	
cortisol 2400	Na	n.s.	0.918	< 0.001	
CgA	K	n.s.		0.019	n.s.	

Abbreviations: NET- neuroendocrine tumor; ACTH—adrenocorticotropic hormone; cortisol 0600—early morning cortisol; cortisol 2400 –midnight cortisol; Na—sodium; K—potassium; P–phosphate; CgA—Chromogranin A; n.s—non-statistically significant

We found that ACTH was significantly positively correlated with Na and negatively correlated with phosphate in EAS patients without NET, whereas there was not any substantial correlation of those electrolytes in patients with NET. Moreover, ACTH considerably negatively correlated with K both in EAS patients with and without NET (as mentioned above). Chromogranin A correlated significantly with K (positively) in EAS patients with NET. EAS patients who died had significantly higher values of early morning cortisol levels (n = 18; 1779.31± 468.97 [nmol/l]) than patients, who survived (n = 6; 1020.69± 965.52 [nmol/l]): p = 0.028) and when assessing patients with and without NET, we observed a significantly higher concentration of midnight cortisol levels (cortisol 2400) in NET patients (p = 0.024). Table 6.

**Table 6:** Data of patients with and without NET.

	total group			female			male		
	NET n = 12	non-NET n = 12	p	NET n = 6	non-NET n = 8	p	NET n = 6	non-NET n = 4	p
K mmol/l	2.65 (2.40–2.81)	2.93 (1.90–3.92)	n.s.	2.40 (1.97–2.50)	2.93 (2.00–4.09)	n.s.	2.81 (2.72–4.50)	2.80 (1.80–3.92)	n.s.
Na mmol/l	145.00 (142.50–148.80)	145.10 (141.00–149.35)	n.s.	146.50 (145.00–149.00)	143.00 (139.00–148.50)	n.s.	143.50 (142.00–148.60)	147.45 (144.10–150.30)	n.s.
Ca mmol/l	2.12 (2.00–2.31)	2.16 (1.83–2.31)	n.s.	2.16 (2.11–2.20)	2.25 (1.89–2.32)	n.s.	2.00 (1.94–2.42)	2.01 (1.64–2.19)	n.s.
P mmol/l	0.90 (0.71–1.21)	0.94 (0.66–1.15)	n.s.	0.94 (0.71–1.18)	0.91 (0.76–1.04)	n.s.	0.85 (0.75–1.21)	0.99 (0.59–1.27)	n.s.
TSH mIU/ml	0.31 (0.11–0.55)	0.38 (0.19–0.70)	n.s.	0.21 (0.03–0.40)	0.45 (0.14–0.80)	n.s.	0.37 (0.15–0.70)	0.29 (0.20–0.38)	n.s.
ACTH pmol/l	75.02 (37.62–98.23)	57.97 (34.10–87.45)	n.s.	82.28 (45,76–107.80)	47.08 (34.10–85.80)	n.s.	70.40 (29.48–75.24)	73.26 (41.69–199.65)	n.s.
cortisol 0600 nmol/l	1779.31 (1531.04–2000.00)	1586.20 (1020.69–1931.04)	n.s.	1779.31 (1655.17–1958.62)	1406.90 (1020.69–1820.70)	n.s.	1806.90 (855.17–2041.38)	1737.93 (1158.62–2068.97)	n.s.
cortisol 2400 nmol/l	1710.35 (993.10–1875.86)	703.44 (441.38–1517.24)	*	1682.70 (1103.45–1765.52)	620.69 (427.59–1434.48)	n.s.	1710.35 (882.76–1986.20)	1227.59 (717.24–1737.93)	n.s.
cortisol 0600 h:2400 h ratio	1.03 (0.98–1.48)	1.23 (1.04–2.80)	n.s.	1.34 (0.98–1.70)	1.78 (1.11–3.33)	n.s.	1.00 (0.97–1.12)	0.94 (0.92–0.95)	n.s.
Testosterone ng/ml	2.50 (2.00–4.00)	4.50 (2.00–7.00)	n.s.	2.00 (2.00–3.00)	3.00 (1.50–5.50)	n.s.	4.00 (2.00–4.00)	12.50 (5.00–20.00)	n.s.
Prolactin μIU/ml	288.50 (218.00–455.50)	262.60 (210.00–422.50)	n.s.	407.00 (223.00–644.00)	289.50 (235.00–508.00)	n.s.	274.50 (218.00–337.00)	234.10 (185.00–283.20)	n.s.
CgA nmol/l	22.50 (4.00–51.00)	5.00 (5.00–12.00)	n.s.	7.00 (2.50–22.50)	5.00 (5.00–12.00)	n.s.	57.00 (51.00–63.00)	----	----

Abbreviations: K—potassium; Na–sodium; Ca-calcium; P–phosphate; TSH—thyroid-stimulating hormone; ACTH—adrenocorticotropic hormone; cortisol 0600—early morning cortisol; cortisol 2400 –midnight cortisol; CgA—Chromogranin A; n.s—non-statistically significant;

*p = 0.024

Furthermore, when assessing patients with different localizations of neuroendocrine tumors separately, those diagnosed with GepNET had statistically higher cortisol 2400 concentrations than non-GepNET patients (median 1765.52 nmol/l and 827.59nmol/l, respectively; p = 0.008). Moreover, GepNET patients had higher ACTH concentrations (median 75.95pmol/l; IQR 41.84pmol/l) than mediastinal NETs (mNETs) (median 45.80 pmol/l; IQR 29.94pmol/l) and other tumors (median 58.03 pmol/l; IQR 51.92pmol/l) and higher cortisol 0600 concentrations and lower potassium levels, although this did not reach statistical significance. Table 7.

**Table 7:** Data of patients with and without GepNET.

Parameter	GepNET	non-GepNET	p
	n = 9	n = 15	
	Med (Q1-Q3)	Med (Q1-Q3)	
ACTH (pmol/l)	75.95 (66.06–107.91)	56.15 (34.13–85.11)	0.34
cortisol 0600 (nmol/l)	1958.62 (1655.17–2041.38)	1655.17 (1089.65–1820.69)	0.13
cortisol 2400 (nmol/l)	1765.62 (1600.00–1986.20)	827.59 (551.72–1517.24)	0.01
K (mmol/l)	2.60 (2.40–2.72)	2.80 (2.00–3.92)	0.47

Abbreviations: GepNET- gastroenteropancreatic neuroendocrine tumor; Med-median; Q1-first quartile; Q3-third quartile; Cortisol 0600- early morning cortisol; cortisol 2400- midnight cortisol ACTH—adrenocorticotropic hormone; K–potassium.

The levels of ACTH did not vary (p = 0.37) in patient with and without distant metastases (median 75.64 pmol/l; IQR 58.30pmol/l vs 45.80 pmol/l; IQR 37.44pmol/l), although if outliers are omitted (1421pg/ml in one case of esthesioneuroblastoma without metastases), the differences become more apparent, though still not significant (75.64 pmol/l; IQR 58.30pmol/l vs 41.95 pmol/l; IQR 24.06 pmol/l in cases without metastases; p = 0.13).

Concerning other laboratory findings: serum calcitonin was elevated in both patients with medullary thyroid cancer, while chromogranin A was elevated in 6 patients: in 4 patients with NET (2 with pulmonary carcinoid and 2 with pancreatic NET) and 1 with pheochromocytoma and 1 with ovarian carcinoma. For 6 NET patients and for 7 non-NET patients there was no data.

Radiological data of the patients is presented in Table 8.

**Table 8:** Radiological data of EAS patients.

	NET	CT ^1^		MRI^1^		Scintigraphy		FDG PET/CT	
Patient No.		Done	Revealed change	Done	Revealed change	Done	Revealed change	Done	Revealed change
1	+	+	+	na	na	+	+	-	-
2	+	+	+	na	na	+	+	-	-
3	+	+	+	na	na	+	+	+	+
4	-	+	+	na	na	na	na	na	na
5^2^	+	+	+	na	na	na	na	na	na
6	+	+	+	na	na	+	+	+	+
7^3^	-	+	+	-	-	+	-	+	+
8	-	-	-	+	+	+	+	+	+
9	-	+	-	-	-	+	+	na	na
10	+	+	+	-	-	+	+	na	na
11	+	+	-	-	-	+	+	na	na
12^4^	-	+	-	-	-	na	na	na	na
13	-	+	+	+	-	+	-	+	+
14	-	+	+	-	-	+	+	na	na
15	-	+	+	-	-	-	-	+	+
16	+	-	-	+	+	-	-	+	+
17	+	+	+	-	-	+	+	-	-
18	-	+	+	-	-	na	na	na	na
19	-	+	+	-	-	-	-	+	+
20	+	+	+	-	-	+	+	+	+
21	-	+	+	na	na	+	-	+	+
22^5^	+	+	-	+	+	+	+	+	+
23	-	+	+	-	-	+	+	+	+
24	+	+	+	na	na	+	+	na	na

^1^conducted in any area of the body;

^2^tumor revealed in gastroscopy before CT performed;

^3^ CT together with PET;

^4^ tumor revealed in the ultrasound of the neck;

^5^CT did not reveal tumor of the pancreas

In most cases, a single imaging study allowed to detect the primary change: In 2 patients, MRI was the first-choice examination, and it was positive, whereas CT was performed in 22 patients, giving a negative result in 4. MRI examination revealed a source of ectopic ACTH in one case. Among 3 patients with negative both CT and MRI–in 2 SRS or FDG-PET revealed a lesion, while the last one had positive ultrasound examination. Concerning CT, hyperplasia of the adrenal glands (AH) was present in 75% of our patients (18 of 24); no data was available for one patient.

In our study, 10 patients diagnosed as NET underwent SRS, in all of those, we observed a high uptake of radionuclide in lesions producing ACTH. Five patients with NET underwent FDG-PET, which was positive. SRS performed in 7 non-NET patients was positive only in 4 cases–all with diffused neuroendocrine system—derived tumors (2 pheochorocytomas, one esthesioneuroblastoma, 1 medullary thyroid carcinoma) confirmed in FDG-PET. In three non-NET patients SRS was negative, while increased glucose metabolism in pathological structures was revealed in the FDG-PET. In 2 patients (ovarian carcinoma and lung tumor), CT (abdominal, pelvic and chest) showed a tumor suspected of malignancy.

In 18 patients MRI of the pituitary was conducted in order to exclude visible pathological mass of the pituitary area.

Concerning treatment, tumor responsible for ectopic secretion was resected in 12 patients (in 2 surgery was not radical), in all patients who underwent radical tumorectomy, the signs and symptoms of Cushing’s syndrome resolved and normalization of cortisol and ACTH levels was observed. Adjuvant treatment included: chemotherapy (1/24), radiotherapy (2/24), Peptide Receptor Radionuclide Therapy (PRRT) and treatment with long-acting somatostatin analogue for all NET patients. To control hypercortisolemia, adrenal steroidogenesis inhibitors (ketoconazole, metyrapone, mitotane) were used. Three patients underwent only palliative treatment due to a poor general condition and two patients: one with MTC and one with thymic NET, underwent bilateral adrenalectomy.

**Fig 2:**
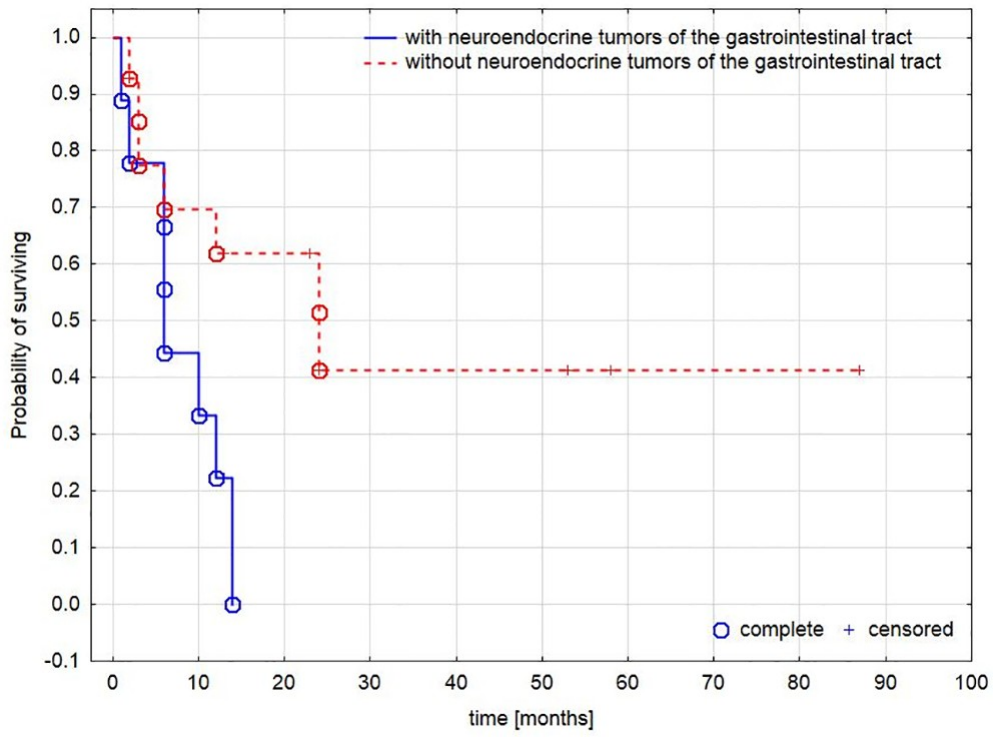
Survival analysis based on type of tumor. The difference in mortality in patients with and without neuroendocrine tumors of the gastrointestinal tract.

Of the whole group of patients, 18 died, most due to widely disseminated disease. Table 1. Among them one patient with co-existing viral hepatitis C died with the symptoms of acute liver failure due to rapid hypercortisolemia and three patients due to complications after surgery; the median duration of follow-up was 8.5 months (range-1-86 months; with a median survival of 9.7 months). Patients diagnosed with GepNET had higher mortality compared to the rest of the group, despite targeted treatment-those were the patients who, at the time of diagnosis of EAS, already had disseminated disease, or had been treated with long-acting somatostatin analogs for many years until progression synchronous with the appearance of EAS. Fig 2.

**Fig 3:**
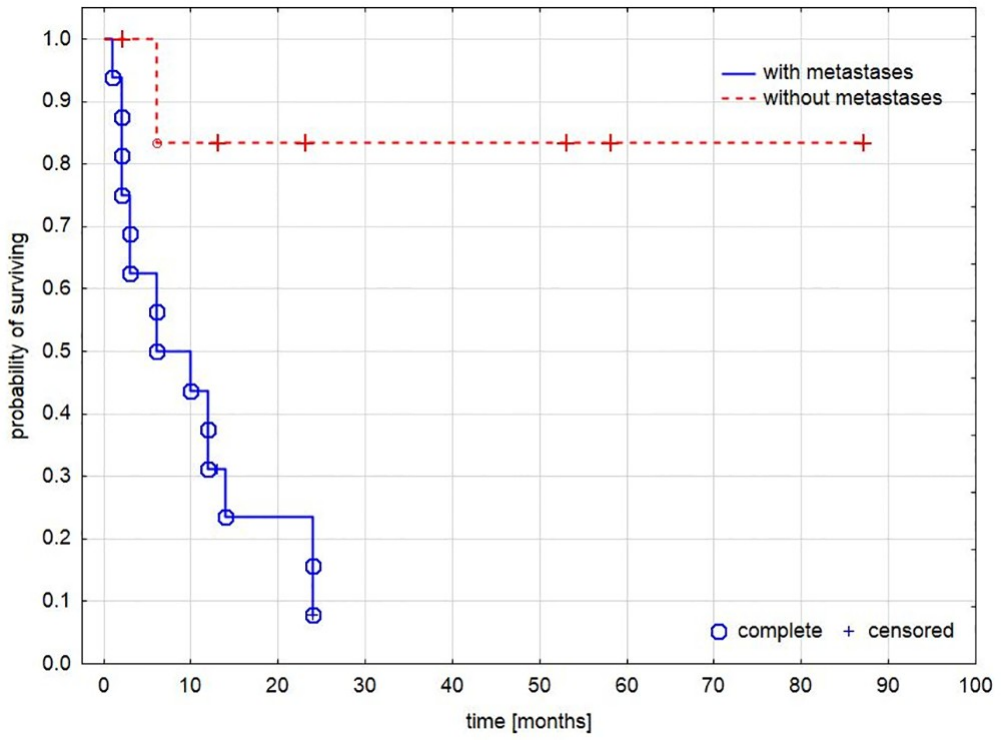
Survival analysis based on type of tumor. The difference in mortality in patients with and without metastases.

Similarly, the presence of metastases significantly worsened the probability of survival. Fig 3.

## Discussion

EAS is a rare disease whose incidence rate is 1 up to 3 new cases/1 million people/year [1, 3–5, 11]. However, if it occurs increases the mortality of patients [8, 15, 16]. It is well known, that hypercortisolemia itself increases the mortality of affected patients. In recently published series, Davi et al. confirmed that the severity of cortisol excess impacts on survival [14]. In our work, patients who died had significantly higher cortisol concentrations than patients who survived.

During our research we focused on EAS in the course of NET and non-NET tumors. We did not compare CD with EAS, but presented a group of patients with EAS focusing on the differences between the course of EAS in NET and non-NET. To our knowledge, no work on the subject has been published so far.

The major finding of our work is the fact that NET and especially GepNET is a special group of EAS patients. As more and more patients with GepNET are being diagnosed recently, more and more EAS cases in the course of NET will appear.

In our study, compared to other tumors, NETs had higher ACTH and cortisol concentrations with lower potassium and TSH levels. Our data are in contrast to those analyzed by Isidori, where NET patients had lower cortisol and ACTH concentrations [4].

Furthermore, when analyzed the GepNET subgroup versus the rest of the tumors -the differences were the highest.

This could be the explanation of another important finding of our study—that GepNET patients have significantly worse overall survival compared with patients with different source of EAS. GepNET patients usually have distant metastases at initial diagnosis [17]. In our group, all GepNET patients had disseminated disease at the time of the diagnosis. On the other hand, it can be partly explained by the fact that the non-GepNET group includes patients with poor prognosis such as carcinomas (ovary, lung, stomach) as well as those with better prognosis such as medullary thyroid carcinoma, pheochromocytoma, esthesioneuroblastoma and patients with lung carcinoid tumors.

ACTH and CRH can be produced by almost all tumors, both malignant and benign, of endocrine and non-endocrine origin [3, 18–23]. In the last decades, we have observed a shift in the prevalence in EAS to more often diagnosed neuroendocrine tumors [8, 19, 23, 24]. Most cases in the first decades after establishing the definition of EAS were caused by small cell lung carcinoma [12, 13, 23]. Still, almost half of the tumors can be found in the thoracic cavity, mostly bronchial carcinoids and SCLCs [4, 8, 11, 24–26]. In our study, on the contrary, most were located in the abdomen or pelvis. Compared with other large studies we observed a higher proportion of GepNETs (37.5% vs 3.0–18.3%) [3, 4, 9, 14, 15, 17, 21, 26]. Table 9.

**Table 9:** Tumors associated with EAS in our study and previously published series.

	Our study 2020	Davi 2017	Ejaz 2011	Doi 2010	Isidori, 2006	Ilias 2005	Aniszewski 2001	Doppman 1989
No of patients (%)	24	110	43	16	40	90	106	28
Mean/ Median age [years]	Mean± SD 56.6±16.3	Mean ± SD 49.5 ± 5.9	Median 49	Mean ± SD 58.4 ± 19.0	Mean ± SD 45.9±15.3	Mean ± SD 37.6±14.8	Mean ± SD 51±18	Average 43
Gender								
• Female	14 (58.0)	64 (58.2)	26 (60.5)	9 (56.2)	19 (50.0)	48 (53.3)	61 (57.5)	19 (67.6)
• Male	10 (42.0)	46 (41.8)	17 (39.5)	7 (43.8)	19 (50.0)	42 (46.7)	45 (42.5)	9 (32.4)
SCLC	1 (4.1)	4 (3.6)	9 (20.9)	2 (12.5)	7 (17.5)	3 (3.3)	12 (11.3)	-
Bronchial carcinoids	2 (8.3)	45 (40.9)	9 (20.9)	3 (18.7)	12 (30.0)	35 (38.8)	26 (24.5)	11 (39.3)
GepNET	9 (37.5)	20 (18.2)	6 (14)	2 (12.5)	3 (7.5)	8 (8.8)	17 (16.0)	3 (10.7)
Other NET	-	-	-	-	4 (10.0)	13 (14.4)	8 (7.5)	-
Thymic carcinoid	1 (4.1)	6 (5.5)	3 (6.9)	1 (6.2)	2 (5.0)	5 (5.5)	5 (4.7)	2 (7.1)
MTC	2 (8.3)	2 (1.8)	5 (1.6)	-	3 (7.5)	2 (2.2)	9 (8.5)	-
Pheo	2 (8.3)	7 (6.4)	-	-	1 (2.5)	5 (5.5)	3 (2.8)	3 (10.7)
Other	5 (20.8)	1 (0.9)	4 (9.3)	2 (12.5)	3 (7.5)	2 (2.2)	-	-
Unknown/Occult	2 (8.3)	25 (22.7)	4 (9.3)	6 (37.5)	5 (12.5)	17 (18.8)	17 (16.0)	9 (32.4)

Abbreviations: SCLC–small-cell lung carcinoma; GepNET–gastroenteropancreatic neuroendocrine tumor; NET—neuroendocrine tumor; MTC—Medullary thyroid carcinoma; Pheo—Pheochromocytoma

This can be partly explained by the fact that in our medical center, we mostly diagnose and treat endocrinological disorders, whereas most patients with SCLC are under care in oncological centers. Another explanation for the different percentage distribution of tumors in our study could be the fact, that those patients do not have time to develop typical cushingoid feature and severe hypokalemia is usually treated symptomatically, without any further diagnostic procedures. Typical cushingoid features are more often seen in latent tumors, compared to malignant neoplasms—in those cases, due to the rapid progression of the underlying disease, typical symptoms of hypercortisolemia may not be revealed. Symptoms of hypercortisolism often appear in advanced stages of non-NET patients, especially in SCLC, when cachexia and electrolyte disturbances dominate which are related to the terminal state of the patient or treatment. Concerning neuropsychiatric disorders (observed in 42% of patients in our group) they are not common in EAS patients, most likely due to a poor general condition, though is some cases it can be the leading symptom of ectopic ACTH production [27–29].

What is more, there was a higher female to male ratio in our study, in contrary to other analyses where there is a male predominance in EAS; probably because most lung cancer patients are males, while GepNET patients are predominantly women [25, 30].

Furthermore, compared to CD, EAS patients are more likely to experience severe hypokalemia, which has been previously broadly studied—the higher the plasma cortisol concentration, the more severe the hypokalemia [14, 25, 31]. Hence, our results are similar. The authors of these earlier publications have proposed an explanation for this phenomenon. They suggested that excessive production of cortisol induces a state in which cortisol itself acts as a mineralocorticoid, regardless of ACTH, saturating the 11beta-hydroxysteroid-dehydrogenase [25]. In comparison to other series- our group had similar prevalence of hypokalemia (70%).

Concerning infections in EAS patients: it is well known that high level of cortisol predispose to infections [32–34]. We have found interesting correlation between the level of hypercortisolemia, predisposition to infections and primary site of tumor responsible for EAS. NET and GepNET patients (with worse prognosis of survival) with infections had significantly higher levels of cortisol. As it was presented in previously reported research–the higher hypercortisolemia, the higher prevalence of infections in affected patients [33].

Our study confirmed that, EAS patients most often had muscle fatigue and hypertension. Similarly, proximal muscle weakness as the leading symptom in the case of hypercortisolemia of ectopic origin is observed in studies from various centers [3, 11, 35–38]. Also well-known is the influence of glucocorticoids on thyroid function (suppression of the hypothalamic-pituitary-thyroid axis) [39–42]. Here, the assessment of TSH by primary care providers can be of great value and practitioners should be aware, that in case of coexisting severe hypokalemia, muscle weakness with low TSH (in patients with no history and signs of thyroid disorders) can strongly suggest hypercortisolemia and EAS. We therefore propose that TSH may be a simple blood test, which could improve the diagnosis of hypercortisolemia.

Concerning imagining techniques, in our work, in all cases the possible source of ectopic ACTH was found in at least one of the imagining techniques. None of the single imaging studies give 100% sensitivity but combining different techniques allows to increase it [7, 43–47]. On top of that, because more and more cases of EAS origin from NETs, SRS begins to play an increasingly important role, especially in the case of negative CT or MRI, and should be taken into account at an early stage of the diagnostic algorithm [45, 48–52]. In EAS non-NET patients with tumors originating from the diffused endocrine system-DES (as esthesioneuroblastoma, pheochromocytoma or medullary thyroid carcinoma), diagnostic procedures can be based on SRS. In those patients, lesions localized in SRS were confirmed by other imaging studies, similarly to NET patients. In other patients with tumors not originating from DES (as carcinomas or papillomas), SRS seems to be of lower diagnostic significance. In those cases, imaging procedures should first be based on CT, MR or FDG-PET. FDG-PET in NETs has a much lower sensitivity in detecting the primary source of tumor [47, 48, 50, 53, 54]. As it was explained by Adams et al., this is mostly due to its limited metabolic activity [55].

In general, hyperplasia of the adrenal glands (AH) is often seen in CS [56, 57]. In our work it was observed in 18 patients (75%), these results are similar to those achieved by Imaki et al., where AH was seen in 75% patients (3 of 4) and in 54% of CD patients [56]. An even higher frequency was reported by Sohaib et al. (90% in EAS patients (9 of 10) compared with 62% in CD patients) [57]. What is common across all studies is that AH was more often seen in EAS patients than in other causes of ACTH-independent CS–probably due to extremely high levels of ACTH in those patients [17, 56, 57].

Confirmation of EAS is challenging. The gold standard in the diagnosis of EAS is BIPSS and confirmation of EAS requires positive staining for ACTH or CRH in tumor cells [4, 10, 20, 54, 58]. In our patients, due to the poor condition of the patients, BIPSS was not available, and in addition, when patients could not underwent tumorectomy, no immunohistochemical evaluation was available.

Pituitary MR examination cannot be used to unequivocally exclude or confirm EAS, because false negative results are also observed in pituitary CS–and it is an important limitation of this method. HDDST, alone, is also of limited value [10]. However, the ACTH concentration in all concerning EAS publications was shown to be significantly higher than in CD patients. Aron in his article has focused on the value of HDDST in the diagnosis of ACTH—dependent CS. He proved that the HDDST has limited value in the differentiation of the source of ACTH—dependent CS. We fully agree with this statement. In our patients, it was one of the tests that in combination with other laboratory tests and clinical presentation allowed EAS to be suspected. What is more, in his publication, Aron showed that compared to CD patients, EAS patients had significantly higher mean ACTH concentration (47 vs 17 vs. pmol/l), as well as a smaller percentage of patients who had suppression by 50% or more of the baseline in HDDST (33.3 vs. 81.0%). Also MR imaging was not a differentiating criterion [10].

The source of ectopic ACTH is neoplastic tissue, which usually is confirmed in immunohistochemistry examination. In our study, only in 3 patients immunohistochemistry staining in the tumor tissue for ACTH was available: in two patients it was positive, and in the other one it was negative. Concerning the last patient, as it was proposed by Isidori and Lenzi, only a subpopulation of cells may actually secrete ACTH which could explain our finding [4].

There are some limits to this study. Firstly, we mainly used archival data. Secondly, the patients came from a single center focused on endocrine diseases. Most of them had at least mild features of hypercortisolemia and/or signs of elevated ACTH. Patients with lung neoplasms are diagnosed and treated in Oncological Centers. Because IPSS considered as the diagnostic gold standard was not available, the diagnosis of EAS was based on laboratory findings, clinical symptoms, imagining techniques and/or resolution of hypercortisolemia after removal of the tumor responsible for EAS. The limitation of this study is also the fact that we failed to distinguish between ectopic CRH and ACTH secretion. Thus, Muller et al. suggested that non-excessive elevation of serum ACTH and a partial response to high-dose dexamethasone test with negative imaging can imply the ectopic production of CRH [59].

## Conclusions

1. The occurrence of hypokalemia in GepNET patients should prompt suspicion of EAS, especially when other symptoms such as hypertension, muscle weakness or weight loss appear.
2. GepNET patients usually do not have time to develop typical cushingoid feature because of rapid progression of EAS.
3. Diagnosis of GepNET in EAS patients significantly worsens the probability of surviving.
4. In active searching for the source of ectopic cortisol production, combining different imagining techniques allows to increase sensitivity. In patients with NET, SRS should be the test of choice.
